# The impact of chronic illness in suicidality: a qualitative exploration

**DOI:** 10.1080/21642850.2014.940954

**Published:** 2014-08-20

**Authors:** Eleni Karasouli, Gary Latchford, David Owens

**Affiliations:** ^a^ Division of Mental Health and Wellbeing, Warwick Medical School, University of Warwick, Coventry, UK; ^b^ Leeds Institute of Health Sciences, University of Leeds, Leeds, UK

**Keywords:** suicide, non-adherence, chronic physical illness, qualitative, grounded theory

## Abstract

*Objectives*: To explore the experiences of patients with chronic physical illness in relation to suicidal behaviours and ideas. *Design*: A qualitative study using semi-structured interviews. *Methods*: Fourteen patients with either multiple sclerosis or stage 5 chronic kidney disease were interviewed. Grounded theory was used to analyse the data. *Results*: Suicidal ideation was commonly reported by the study participants, and the relationship between the impact of a chronic physical illness, suicidality and risk factors was described. Several participants reported having planned suicide attempts as a consequence of finding living with their illness intolerable, and some had used non-adherence to treatment as a deliberate method to end their life. *Conclusion*: The findings suggest suicidality may be a relatively common experience in those with chronic illness facing a future of further losses, and that alongside passive thoughts of not being alive this may also include active thoughts about suicide. Health professionals should be alert to intentional non-adherence to treatment as an attempt to end one's life.

## 1. Introduction

The study of suicidality (completed suicide, non-fatal attempts or self-injurious behaviours and ideas of suicide) has understandably focused largely on the psychiatric characteristics associated with it. Stressful life events are known to play a role in increasing risk however (Stein et al., 2010), and this has led to an interest in the potential role of chronic physical illness.

For many, chronic physical illness is associated with decreased quality of life and a number of practical, psychological and social problems (Berman & Pompili, 2011; Sidell, 1997; Wikman, Wardle, & Steptoe, 2011). Depending on the condition, patients may suffer from pain or other symptoms, disability, disfigurement and an invasive treatment regimen. Chronic illness can affect the ability to participate in work or leisure activities leading to social isolation, and is associated with increased rates of anxiety and depression. For those individuals that struggle with their illness, increased suicidality appears to be a very real possibility. Available evidence so far shows a link between suicide and a number of medical conditions, including stroke (Bronnum-Hansen, Davidsen, & Thorvaldsen, 2001; Pompili, Venturini et al., 2012; Teasdale & Engberg, 2001), HIV/AIDS (Keiser et al., 2009; Marzuk et al., 1997; Roy, 2003), stage 5 (end stage) chronic kidney disease (stage 5 CKD) (Kurella, Kimmel, Young, & Chertow, 2005) and multiple sclerosis (MS) (Bronnum-Hansen, Stenager, Stenager, & Koch-Henriksen, 2005; Fredrikson, Cheng, Jiang, & Wasserman, 2003; Pompili, Forte et al., 2012). Thus far, however, the mechanisms underlying this relationship have not yet been explored.

A first step towards understanding this is to explore the experiences of suicidality of patients with these chronic illnesses. In the current study, two chronic medical conditions, known to be associated with increased risk of suicide, MS and stage 5 CKD, were chosen to explore the relationship with suicidality. Both conditions are life-limiting, intrusive in terms of activities of daily living, and have a major impact on quality of life. Given the relatively unexplored nature of the topic, a qualitative approach was chosen to address one broad research question: what are the experiences of suicidality of patients with MS and stage 5 CKD?

## 2. Method

### 2.1. Participants

Inclusion criteria were diagnosis of MS or stage 5 CKD; age between 18 and 75 years; and ability to speak English. All of the participants with stage 5 CKD were on haemodialysis. Though there is clearly a link between depression and suicidality in the mental health literature, we did not want to make any assumptions about this in physical health. We were also mindful that a physical health setting might not be conducive to a disclosure of either depression or thoughts of suicide, so that services may not necessarily be aware when these are present. We therefore chose not to specify history of depression or suicidal ideation as inclusion criteria.

### 2.2. Procedure

Consultant neurologists and renal physicians in a large teaching hospital in the UK recruited potential participants during routine outpatient consultations. The sample was a purposive one and any patients with either of the two physical conditions of interest were eligible to take part in the study. Contact was later made by the primary investigator (E. K). who consented participants into the study if they were willing to take part. E. K., a trained researcher in qualitative methods, interviewed all participants. All interviews took place in the participants' homes, and were completed in a single visit. Interviews lasted between 45 and 180 minutes. Participants completed a demographic questionnaire at the beginning of the interviews. Due to the sensitive subject area of the interviews, a general question on patient satisfaction with the services was incorporated at the end of the interview schedule to give the opportunity to participants to ‘wind down’. All participants were offered the opportunity of debrief or therapeutic support independent of the interviewer. Interviews were audio recorded and later transcribed by a professional transcriber. The study received ethical clearance from the Leeds West NHS Research Ethics Committee.

### 2.3. Measures

Participants completed a self-report demographic questionnaire. This was designed for the study and gathered information on the participants' medical condition and general demographics.

### 2.4. Interviews

A semi-structured interview schedule was developed after a review of the literature, and after consulting a number of health professionals working with patients with MS and stage 5 CKD. The interview schedule featured open-ended questions around five domains: experience of diagnosis, physical health, mental health, perception of stressors and adjustment. The topics focused on the experiences of having a chronic illness, particularly factors which participants perceived as being more or less stressful. As part of this, all participants were asked whether there were times when they felt they had managed well, and whether there were times when they had experienced suicidality. During the semi-structured interviews the interviewer used a conversation-like method in which she was guided by the interview schedule, but the conversation could vary between participants. This method allowed the participants to focus on what they thought was important.

### 2.5. Analysis

Data collection and analysis was guided by a grounded theory (GT) approach (Strauss & Corbin, 1990). A number of qualitative methods were initially thought suitable; however, GT was more appropriate due to its theory-building aspect. For example, approaches such as the framework analysis use a more deductive form of analysis in which the aims and objectives of the study are set in advance rather than emerging from the reflexive research process (Pope, Ziebland, & Mays, 2006). Thus, the topic guide used for the interviews in order to collect data is usually more structured than is the norm for most qualitative research. As part of the GT approach, a number of steps were followed in moving from the collection of unstructured data through to the emergence of theory. Data were read and re-read for the initial coding, followed by the development of categories and then of a more sophisticated level of coding. Study templates were used from Charmaz (2006) and Straus and Corbin (1990) for open, axial and theoretical coding, using an iterative process rather than a linear method, as suggested by Glaser and Strauss (1967). E. K. was responsible for initial coding and data analysis with continuous consultations between all authors. NVIVO was used to store and organise the data.

### 2.6. Rigour

As suggested by Elliott, Fischer, and Rennie (1999), coding credibility checks were carried out to replicate the process of coding and calculate percentage of agreement. Two independent coders were given 20 interview quotes to match with the relevant subcategory. Coder 1 correctly matched 20/20 of the quotes. Coder 2 matched 18/20. In addition, participant validation was used to check the theoretical formulation (Miles & Huberman, 1994). All participants were sent a copy of the preliminary theoretical formulation and asked for their feedback on how well it matched their own experience. Replies were received from five, and all were in broad agreement with the model.

## 3. Results

### 3.1. Participants

Fourteen participants were interviewed; six were diagnosed with MS and eight with stage 5 CKD. Seven were females and seven were males. Ages ranged between 22 and 66 years. The participants' details are shown in Table 1.

**Table 1.** Participant information.

Interviewee	Medical condition	Age	Gender	Education	Employment status	Marital status	Comorbidity
P.01	MS	43	Female	High school or equivalent	Employed	Married/living with another	No
P.02	MS	50	Female	Vocational/technical school	Unemployed	Married/living with another	No
P.03	Stage 5 CKD	66	Male	High school or equivalent	Retired	Married/living with another	Yes
P.04	Stage 5 CKD	57	Male	Bachelor's degree	Employed	Married/living with another	Yes
P.05	Stage 5 CKD	22	Female	High school or equivalent	Unemployed	Single	No
P.06	MS	56	Female	College	Retired	Married/living with another	No
P.07	Stage 5 CKD	47	Male	College	Unemployed	Married/living with another	No
P.08	MS	46	Female	Bachelor's degree	Retired	Married/living with another	No
P.09	Stage 5 CKD	61	Male	Grammar school	Employed	Married/living with another	Yes
P.10	Stage 5 CKD	51	Male	College	Unemployed	Widower	No
P.11	MS	50	Female	Bachelor's degree	Employed	Separated/divorced	No
P.12	Stage 5 CKD	48	Male	High school or equivalent	Employed	Married/living with another	Yes
P.13	Stage 5 CKD	62	Male	Missing	Retired	Married/living with another	Yes
P.14	MS	51	Female	Bachelor's degree	Employed	Married/living with another	No

### 3.2. Qualitative results

Using GT, the relationship between suicidal behaviour/ideation and coping in chronic physical illness was described, highlighting risk factors. Suicidality is linked with unsuccessful coping in the face of the challenges presented by living with a chronic illness, particularly the prior adoption of denial.

#### 3.2.1. The challenge of physical illness

The participants described in detail the physical, social and emotional consequences of living with a chronic illness. All participants described their reactions to the initial diagnosis and early onset of symptoms, but for most later physical deterioration, either experienced as a sudden onset of new symptoms or as a gradual process, was particularly difficult to manage, often leading to suicidal thinking. This was especially true for those who persisted to use their previous healthy self as a reference point.

I used to be a keep fit fanatic, training, football, rugby, running, walking, climbing, I did the lot. Now walking is a hassle, anything else is a hassle. Walking I can just about get up to the shop, you know, and back, that's about it. And then I have to stop a couple of times to get up there. I used to walk 10–15 miles a day with my partner like at weekends when I wasn't working.

If I'd have got worse then I wouldn't have seen the point.

Even though participants tried to stay active in the first stages of physical deterioration, they reported that it gradually became difficult to keep to their previous pace. Fatigue was frequently mentioned as a reason for the decline in physical activity, despite a willingness to do more, and this also had an emotional impact.

I always try and keep as active as I can, because you know, like going out and things like that helps you, going for a walk a day, but the fatigue just gets me, that really does get me down actually at times. And I find that very hard, so that's the horriblest thing for me at the moment. And the fact that I can't do as much as I want to do does get me down. So I don't quite know how to get through that a lot of the time. And I do sit on there and cry, or lie on there and um, and have a good sob, yeah.

The physical challenges of chronic illness were often linked to social and emotional consequences, with the condition impacting on all aspects of the participants' lives, interfering with employment, leisure and social activities. The participants understood this impact in terms of the illness limiting their choices, and limiting the amount of control they experienced in their life. Some discussed how the rules about living that they learned throughout life no longer applied. The participants were particularly mindful of the effects on family and the increased dependence on others. This in turn impacted on role satisfaction and self-esteem, leading to increased suicidality.

One of the things that used to affect me … well it still affects my self respect that I can't go out to work.

I really wanted out, well in fact I attempted suicide … it costs £25,000 a year to keep me alive plus whatever amount of money it costs for the drugs and whatever I get on benefits. I've never had benefits in my life up until I came here, never been on the dole in my life. I've always worked all my life, I've had my own business for god knows how many years.

#### 3.2.2. Risk factors

Some factors discussed by participants appeared to make hopelessness and suicidal thoughts more likely. All participants reported struggling with the chronic and incurable nature of their medical condition at times. Metaphors used to describe managing their illness included ‘a continuous battle’ and ‘a never-ending set of challenges’. One consequence is that it is difficult to retain hope for the future, with the prospect of decline and further loss leading to loss of confidence and low mood. This in turn leads to further retreat from social activities.

I don't sort of put myself out anymore, you know, because of the way I feel about myself, the way I look. You know, even when I look at myself I cry so what's another woman going to think like, you know. So I just don't bother any more.

Some participants described this leading to a stage where they felt like ‘giving up’ or ‘resigning’, hope having run out. This was associated with thoughts about suicide. Though this included passive ideation, such as imagining not waking up in the morning, more active suicidal ideation was also reported.

One common response to these challenges was an active refusal to accept the condition and engage in adaptation; participants instead reported that they opted to deny the impact of the illness and attempt to live their life as if they were well. This often meant ignoring medical advice, pushing their body ‘to the limits’.

Um, when I started on dialysis the second time … particularly with the fluid restrictions I found it difficult to stick to them. I never came in with so much fluid on me they couldn't dialyze it all off, but quite often I were at the limit of what we could dialyze off, and that's not good.

For some denial was present at the initial diagnosis, for others denial was reasserted as a dominant coping strategy at different stages throughout the illness.

Some participants talked about denial in terms of not being able to accept the condition and its implications. Some maintained this stance despite reminders of the reality of their illness triggered by the treatment on which they depended. This was particularly true of those on dialysis.

To say, well actually I didn't … I always thought I accepted MS very good and laugh about it, but I hadn't at all, I hadn't accepted it one little bit.

Denial was not successful in the long term, and difficult to maintain in the face of further physical deterioration in the health condition, which in turn triggered low mood and loss of hope.

#### 3.2.3. Suicidal ideation and behaviour

The previous sections present the difficulties that participants encountered which led them to suicidal thoughts or behaviour. Most of the participants reported that at least one point during their time living with the illness they were struggling to cope to such a degree that they saw ending their lives as a possible solution to their problems. These thoughts were linked to the severity of their symptoms, the perceived burden of the treatment and ruminations about the incurable nature of their condition. Some were clear that they had made active plans to take their own life.

I have less of a life, so I just think that, when things are getting bad, I just think that I'd be better off if I wasn't … it may be better if I wasn't here.

Suicidal ideation was common among the participants, and for some there had been a move from ideas to actions that involved considering suicide, planning the method of ending their life and instigating the action. For some, it was a single occurrence whereas for others it was a repetitive process.

And another time I was out and I was going to just drive the car really fast into a wall, just to finish myself off.

Two participants described active plans that they had made to take their own life.

At that time I did feel like ending it, yeah, being back on dialysis after about a year or so, yeah. You think … I'm not even on the transplant waiting list now … I weren't feeling so good, things like that. And yeah I did have those feelings.

I just couldn't do anything, and I remember one time I was going to take some pills to finish it all off because things just got so bad.

The participants with stage 5 CKD in the current study were aware that non-adherence to dialysis would mean that they would effectively be ending their lives, and several reported that they had become non-adherent with this specifically in mind, and without discussing this with their medical team.

I gave it [dialysis] up for ten, eleven, maybe twelve days and I was very ill after it and my neighbour found me on the floor collapsed.

## 4. Discussion

Though many adapt well to the physical and psychosocial demands of living with a chronic illness, there are times when they struggle, and the current study highlights the potential for the experience of suicidality during these times. The two conditions in the study – MS and stage 5 CKD – as many chronic conditions – are associated with an accumulation of losses, and participants described reaching points in their coping when they felt that they were unable to carry on. Sometimes, this was also associated with prior coping strategies that were ultimately unhelpful, such as denial.

Some participants described an active refusal to accept their condition, denying the implications and avoiding reminders so as to maintain their previous way of living. Denial as a coping strategy is known to be associated with non-adherence (Nichols, 1993) and poor adjustment (Noy et al., 1995), but though it is not related to the nature or severity of the medical condition, it is negatively associated with duration of illness. In the current study, denial at diagnosis or in the early stages of the disease was described, but there was also the suggestion that failure to accept the condition may linger on at a deeper level. Kaba et al. (2007), in a study of patients with stage 5 CKD, found an association between struggling to cope, the expression of denial and thoughts of suicide.

An influential model of suicidal behaviour which looks at the underlying psychological mechanisms, referred to as the ‘cry of pain’ model (Williams, 1997), views suicidal behaviour as an attempt to escape from a feeling of entrapment. According to the model:

Particular stress events that precede suicidal behaviour are those that signal defeat; the particular psychological processes that increase vulnerability are those that signal that there is no escape … ; and the particular psychological processes that turn a crisis into a suicidal crisis are those that signal no rescue, increasing hopelessness through, for example, biased judgments of the future. (Williams & Pollock, 2000, p. 80)

Relating this to the current study, the chronic medical condition, its associated symptoms and treatment are stress factors that may well lead the individual to feel trapped. The illness is incurable and hence feelings of vulnerability and lack of escape or control may arise, as noted by some of the participants in the current study. This may lead to suicidal behaviours as a ‘cry of pain’. The model emphasises not only personal factors, but also the importance of life events in triggering such behaviours. According to Kerkhof and Arensman (2001), vulnerability to suicidal behaviours and ideation may be an underlying trait in many people. However, this trait may not be persistent throughout the whole of one's life. The level of this vulnerability and thus, appearance of prominent suicidal behaviours, varies from time to time, depending on the course of life and on subjective thresholds of tolerance. This vulnerability has to be triggered to become manifest, as in the case of a chronic medical condition.

Suicide planning was reported by several participants, who progressed to varying stages in acting upon them, sometimes more than once. One unexpected finding was that non-adherence was reported by patients with stage 5 CKD as being seen, at times, as a deliberate attempt at suicide rather than a problem maintaining adherence. One participant reported a previous, serious attempt at suicide by missing dialysis. This finding echoes that of Haenel, Brunner, and Battegay (1980), who found that 8 out of 10 Swiss patients with stage 5 CKD on dialysis choose non-adherence as a method for ending their lives. This, however, is based on a single study and warrants further investigation.

There is inconsistency among previous studies on whether non-adherence or even withdrawal from treatment in patients with stage 5 CKD should be considered a suicide act (Kimmel, 2001; United States Renal Data System, 2004). Although patients with stage 5 CKD can easily die deliberately through non-adherence (Kimmel, 2001; Kurella et al., 2005) with a rate sometimes reaching up to 84% higher than in the general population, it is argued that actual suicidal behaviour and ideation might be difficult to differentiate in this population (Cohen, Germain, & Poppel, 2003; Kimmel, Weihs, & Peterson, 1993; McDade-Montez, Christensen, Cvengros, & Lawton, 2006). The experiences of the participants in the current study, however, would suggest that non-adherence to treatment can clearly be linked with a desire to die, at least in some individuals. Our participants were able to articulate the factors that had preceded their decision, such as the fear that they had become dependent on others, and the thought that had gone in to the choice of method.

Physical deterioration and the losses associated with this was reported by some participants to have contributed to their thoughts about suicide and attempts to bring about an end to their lives. Previous research on MS and suicide ideation has also highlighted this association, finding that three particular aspects of physical disability (decreased mobility, decreased bowel function and decreased bladder function) played an important role (Turner, Williams, Bowen, Kivlahan, & Haselkorn, 2006). Overall, it has been evidenced that functional limitation is a better predictor of suicide than the illness per se (Kaplan, McFarland, Huguet, & Newsom, 2007).

In the current study, the perception of being dependent on others – particularly family members – was also raised by participants as a major concern. When chronic illness requires changes in the patients' lives, those changes clearly often impact on the wider family. Patients are aware that they may lose their autonomy, and this may be associated with psychological sequelae such as loss of self-esteem and guilt at being a burden on their families. This may lead to thoughts that others would be better off if they were dead. Joiner (2005) has argued that suicide seems to be a solution for those who are physically disabled and see themselves as a constant burden to others, especially their close kin.

Inability to cope with the illness and its symptoms was discussed in relation to suicidal behaviours or ideation. The participants perceived the illness as such a burden to their lives that they did not know how to manage it, sometimes feeling overwhelmed. This perhaps can be explained with reference to Neuringer's (1976) assumption that people who are suicidal are more rigid in their way of thinking than non-suicidal people. Even though most people have the flexibility to moderate dichotomies, those that are suicidal, it is argued, do not have this capacity. This means that the suicidal person will find it difficult and sometimes impossible to modulate their expectancies and to imagine compromise when faced with a problematic situation. Thus, they will perceive the situation as offering few opportunities for relief or change. This supports the ‘cry of pain’ model in that there is no escape or rescue. When the individual is unable to foresee an escape method, they believe that nothing will change in the future and they feel vulnerable and hopeless. As with the chronicity and incurability of medical conditions such as MS and stage 5 CKD, the individual may think that they cannot change the situation they are in and hence engage in suicidal behaviours and ideation.

### 4.1. Implications

The findings of this study suggest that health professionals caring for patients with chronic medical conditions need to be alert to the possibility that patients may experience episodes of suicidal thinking, and that this may lead to active attempts to end their lives. For some, this may include deliberate non-adherence to medication or treatment with an intent to take their own life. Health professionals should be better prepared to monitor and discuss suicidality with their patients.

Apart from the availability of ending dialysis as a means to end life, the current study did not identify any significant differences in the experiences of participants with either of the two medical conditions. It may therefore be suggested that the struggles with coping and the presence of suicidal ideation may be a factor in adaptation to other serious chronic medical conditions. To date, little attention has been given to the relationship between physical illness and suicidal behaviour or ideation. Though longitudinal studies are required to plot the interrelationship between suicidality and adaptation, this study adds to the debate by highlighting the experiences of people with chronic illness of suicidal ideation and suicidal behaviour that has not led to completed suicide, suggesting that it may be a relatively common part of the experience of coping.

### 4.2. Conclusion

This study suggests that suicidality and the desire to end life, either directly through suicide or indirectly through non-adherence, may be an important consideration for patients trying to cope with a chronic illness. At a time when medical advances are leading to people with chronic illnesses living longer, it is important for health services to be aware of the risk of suicidality in their patients.
